# S100A12 as a key biomarker in a neutrophil-associated gene prediction model for sepsis diagnosis

**DOI:** 10.1097/MD.0000000000046140

**Published:** 2025-11-21

**Authors:** Xiaoping Huang, Zibo Yu, Zhifang Zhuo

**Affiliations:** a Department of Anesthesiology, The First Hospital of Putian City, Putian, Fujian, China.

**Keywords:** machine learning, Neutrophils, S100A12, sepsis, SHAP, single-cell RNA sequencing

## Abstract

Sepsis is a life-threatening organ dysfunction syndrome caused by a dysregulated host response to infection. As a leading cause of mortality in intensive care units patients, it still lacks sensitive biomarkers. Therefore, this study aimed to develop a diagnostic model for sepsis and identify key driver biomarkers. Using single-cell RNA sequencing (scRNA-seq) data from the GEO database, we constructed a diagnostic model through 113 machine learning (ML) frameworks, supplemented with Shapley additive explanations (SHAP) analysis to identify pivotal genes. Results revealed a significant increase in myeloid cells, particularly neutrophils, in the peripheral blood of sepsis patients. Screening identified 70 upregulated and 762 downregulated neutrophil-associated genes, which were intersected with differentially expressed genes (DEGs) between healthy controls and sepsis patients, yielding 13 overlapping genes – including S100A12 – as potential drivers. These 13 genes were incorporated into 113 ML models. The Random Forest (RF) model, which included S100A12, PIK3AP1, HLA-DMB, and RETN, achieved the highest mean C-index with fewer features. Its robust diagnostic performance was validated using receiver operator characteristic curves, calibration curves, and decision curve analysis. SHAP analysis highlighted S100A12 as the most influential driver gene and identified theophylline, aspirin, and aminophylline as potential targeting compounds. In conclusion, sepsis patients show increased peripheral neutrophils, an RF model based on 4 neutrophil-associated genes demonstrates strong diagnostic ability, and S100A12 serves as a key biomarker for sepsis.

## 1. Introduction

Sepsis is a life-threatening organ dysfunction syndrome caused by a dysregulated host response to infection, claiming over 11 million lives globally every year.^[1,2]^ As the leading cause of death in intensive care units, its management remains primarily supportive, with measures including antibiotics and fluid resuscitation.^[3,4]^ Due to the lack of mechanism-targeted interventions, sepsis-associated mortality persistently ranges from 10 to 16%.^[5]^ The complex interplay among pathogens, inflammatory cascades, and immunoparalysis underlies pathophysiological heterogeneity.^[4,6,7]^ Consequently, characterizing the heterogeneity of cellular components in the peripheral blood of sepsis patients represents a critical focus for exploring sepsis pathogenesis.

During sepsis pathogenesis, neutrophils function as a critical double-edged sword. As core effector cells of the innate immune system, they are critical for pathogen clearance; however, when hyperactivated or dysfunctional, they activate multiple pathways that exacerbate multi-organ injury in sepsis.^[8,9]^ Prior studies have shown that neutrophils can induce immunometabolic dysregulation via lactate-mediated CD31^+^-dependent migration pathways, thereby exacerbating neuroinflammation in sepsis-associated encephalopathy^[10]^; alternatively, activation of platelet-specific STING can mediate platelet activation and granule secretion, consequently triggering sepsis-induced intravascular thrombosis and neutrophil extracellular trap formation (NETosis) in mice, which synergistically promotes the onset and progression of sepsis.^[11]^ Nevertheless, the potential clinical value of neutrophils in sepsis diagnosis and therapy, along with their associated molecular regulatory networks, remains to be fully elucidated.

Currently, sepsis diagnosis primarily relies on blood cultures and serum inflammatory markers, including white blood cell count, as well as serum inflammatory markers such as C-reactive protein (CRP) and procalcitonin (PCT).^[12,13]^ However, blood cultures, as the diagnostic gold standard, suffer from insufficient timeliness, while serum inflammatory markers exhibit lower sensitivity and specificity.^[14,15]^ In recent years, machine learning (ML), with its superior predictive capabilities, has emerged as a prominent tool for developing disease diagnostic models.^[16–18]^ Multiple studies have developed ML models by utilizing clinicopathological data to accurately predict sepsis onset and prognosis. With advances in sequencing technologies, the use of multidimensional data has become an inevitable trend in medical research. In this context, ML enables the integration of complex multidimensional data to build predictive models. Concurrently, Shapley additive explanations (SHAP) analysis addresses the “black box” limitation of ML models, precisely quantifying each variable’s contribution to sepsis development and delineating synergistic interactions among variables.^[19–21]^ This approach enables data-driven quantification of the regulatory roles of core genes, providing a quantitative basis for systematically identifying key regulatory factors in sepsis and elucidating their roles in immune dysregulation.

Therefore, in this study, we integrated single-cell RNA sequencing (scRNA-seq) data and bulk transcriptome data from sepsis patients and healthy individuals. By analyzing differentially expressed genes (DEGs) across these cohorts and constructing a diagnostic model via ML frameworks, we aimed to achieve precise diagnosis of sepsis onset. Finally, SHAP analysis was applied to elucidate key sepsis biomarkers, providing a scientific foundation for subsequent sepsis research.

## 2. Materials and methods

### 2.1. Single-cell data

The single-cell data used in this study were downloaded from the GEO database with the ID GSE175453. This dataset comprises single-cell sequencing data of circulating immune cells obtained from 4 sepsis patients and 5 healthy subjects. Whole blood samples were collected via venipuncture from patients in the late phase of sepsis (days 14–21) who met the Sepsis-3 criteria, as well as from healthy control participants. 10 × scRNA-seq data were processed according to the following steps with reference to previously published literature^[22]^: the 10 × scRNA-seq data were converted into Seurat objects using the “Seurat” package in R software, and batch effects were removed using the “harmony” package; low-quality cells were removed using the criteria “nFeature_RNA > 200 & percent.mt < 20”; the top 1500 highly variable genes were screened using the “FindVariableFeatures” function; principal component analysis was performed based on the 1500 highly variable genes, followed by dimensionality reduction and clustering using UMAP; important marker genes were identified using the “FindAllMarkers” function; different cell clusters were annotated based on marker genes from the CellMarker 2.0 website (http://bio-bigdata.hrbmu.edu.cn/CellMarker/index.html) and Cell Taxonomy (https://ngdc.cncb.ac.cn/celltaxonomy).

### 2.2. Bulk-RNA data

The bulk-RNA data used in this study were downloaded from the GEO database, including GSE28750, GSE46955, and GSE69528. Among them, GSE69528 comprises 55 healthy subjects and 83 sepsis patients, with blood samples collected within 24 hours after sepsis diagnosis; GSE28750 includes 10 healthy subjects and 20 sepsis patients, with blood samples obtained within 24 hours following surgery; GSE46955 consists of 28 healthy subjects and 16 sepsis patients, with blood samples collected within 2 to 4 hours after admission. Detailed clinical information is provided in Table S1 (Supplemental Digital Content, https://links.lww.com/MD/Q734).

### 2.3. Differential analysis

For single-cell data, the “FindAllMarker” function of the Seurat package was utilized: with neutrophils as the target population, genes were screened by comparing with other cells, and those with |logFC|>1 and *P* < .05 were defined as neutrophil-associated genes. For transcriptome data, using sequencing data from healthy subjects as controls, genes meeting the criteria of |logFC| > 1 and *P* < .05 were screened via the limma package as DEGs. Finally, volcano plots were generated using the ggplot2 package to visually display the degree of expression difference and significance of DEGs.

### 2.4. Enrichment analysis

GO and KEGG enrichment analyses of DEGs were performed using the clusterProfiler package in R programming language. GO analysis covers molecular function, cellular component, and biological process, while KEGG analysis focuses on signaling pathways, with the entire genome as the background. P-values were calculated via hypergeometric tests, adjusted using the Benjamini–Hochberg method, and significantly enriched terms with *P* < .05 were selected. Visualization results such as bubble plots were generated using the enrichplot package to reveal the biological functions and involved pathways of DEGs.

### 2.5. Diagnostic model construction

Feature genes were screened using 113 algorithm combinations with reference to existing literature to construct diagnostic models.^[23]^ This method incorporates 12 ML algorithms, including Random Forest (RF), Least Absolute Shrinkage and Selection Operator (Lasso), Ridge, Elastic Net (Enet), Stepglm, Support Vector Machine (SVM), glmBoost, Linear Discriminant Analysis (LDA), Gradient Boosting Machine (GBM), eXtreme Gradient Boosting (XGBoost), and NaiveBayes. Specific steps for model construction are as follows: expression data of overlapping genes between transcriptome and neutrophil-associated genes were selected; the GSE69528 cohort was used as the training set, with GSE46955 and GSE28750 as validation sets, and the C-index of each ML method in different datasets was calculated separately; the model with the highest mean C-index and the smallest number of genes was regarded as the optimal model.

### 2.6. Model evaluation

The predictive performance of the model was validated using calibration curves and decision curve analysis (DCA) curves. Calibration curves evaluate the consistency between predicted values and actual values by comparing the model’s predicted probabilities with actual observed results. DCA curves are used to evaluate the clinical utility of the model.

### 2.7. SHAP analysis

SHAP value modeling was employed to conduct interpretive analysis of the ML predictive model. Specifically, A SHAP summary plot was generated to visually display global feature importance, ranking features by the mean absolute SHAP value to present their overall impact. Dependency plots were used to analyze the marginal effects of individual features across different value ranges on prediction outcomes, and force plots were employed to explain the prediction logic for individual samples, clearly illustrating how features collectively contribute to the final predicted value. Through SHAP analysis, we not only evaluated the importance ranking of features but also delved into the internal decision-making logic of the model, enhancing its interpretability and credibility.

### 2.8. Potential drug analysis

Collect the 3D structure of compounds from the PubChem website (https://pubchem.ncbi.nlm.nih.gov/), retrieve protein IDs from the UniProt database (https://www.uniprot.org/), input the retrieved IDs into the RCSB PDB database (https://www.rcsb.org/), and download the protein structures. Then use Autodock.vina software for docking to obtain the binding energy. A binding energy of <−5 indicates that the 2 can bind well. Finally, use Discovery Studio software for visualization.

### 2.9. Statistics

All statistical analyses and visualizations were performed using R 4.1.0 software. Continuous variables were summarized as mean ± standard deviation (SD), and categorical variables were summarized using counts or percentages (%). The Chi-square test was used to compare frequencies among categorical variables. Wilcoxon rank-sum test or t-test was used to compare differences in continuous variables between 2 groups. Receiver operator characteristic curve analysis was performed to evaluate binary classification performance. Two-tailed *P*-values < .05 were considered statistically significant.

### 2.10. Ethical approval

The data for this study were obtained from previously published studies and public databases; therefore, no ethical approval was required.

## 3. Result

### 3.1. Single-Cell differences in peripheral blood of healthy and sepsis patients

**Figure 1.**
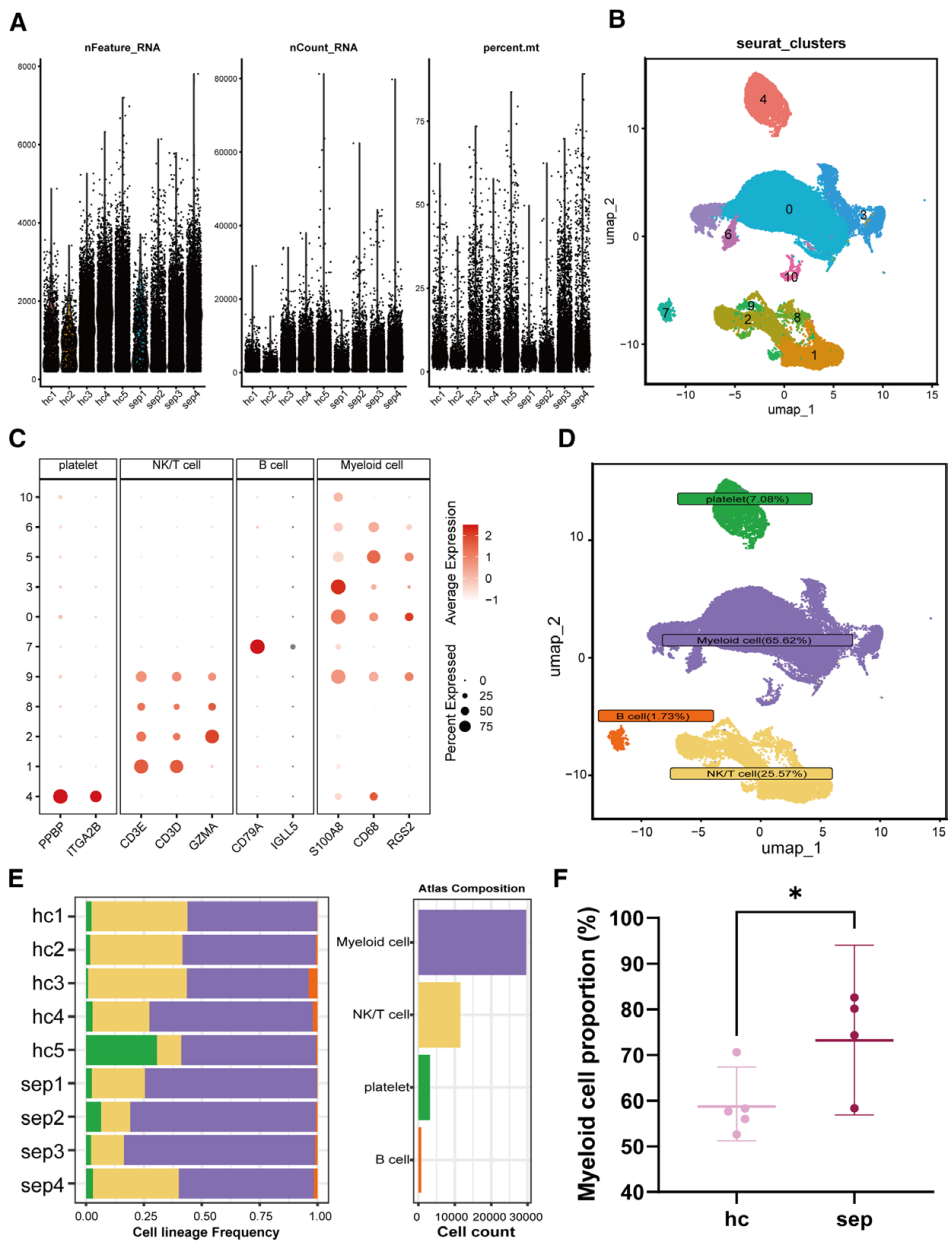
Single-cell profiling of peripheral blood in healthy individuals and sepsis patients. (A) Distribution of per-cell metrics (nFeature_RNA, nCount_RNA, percent.mt) in the scRNA-seq dataset GSE175453. (B) UMAP visualization showing 11 cell clusters. (C) Dot plot displaying marker gene expression across clusters. (D) UMAP plot annotated with cell identities. (E) Bar plot showing proportions of the 4 major cell types. (F) Comparison of myeloid cell proportions between the 2 cohorts. * *P* < .05.

The analysis incorporated scRNA-seq data from 5 healthy subjects and 4 sepsis patients. Figure 1A displays per-cell metrics in the single-cell dataset: number of detected RNAs (nFeature_RNA), total RNA counts (nCount_RNA), and percentages of mitochondrial genes (percent.mt). After filtering low-quality cells, 45,160 cells were retained. Following batch correction with Harmony, dimensionality reduction and clustering were performed, yielding 11 distinct subpopulations (Fig. 1B). These 11 subpopulations were annotated into 4 cell types based on marker genes: Platelets (PPBP, ITGA2B; 3199 cells, 7.08%), NK/T cells (CD3D, CD3E, GZMA; 11,546 cells, 25.57%), Myeloid cells (S100A8, CD68, RGS2; 29,632 cells, 65.62%), and B cells (CD79A, IGLL5; 783 cells, 1.73%) (Fig. 1C and D). Additionally, analysis of cell proportions across patients revealed that the proportion of Myeloid cells was significantly increased in sepsis patients (*P* < .05; Fig. 1E and F), indicating that the differentiation and recruitment of Myeloid cells in the blood play a key role in sepsis development.

### 3.2. Differences in myeloid cells between sepsis patients and healthy individuals

**Figure 2.**
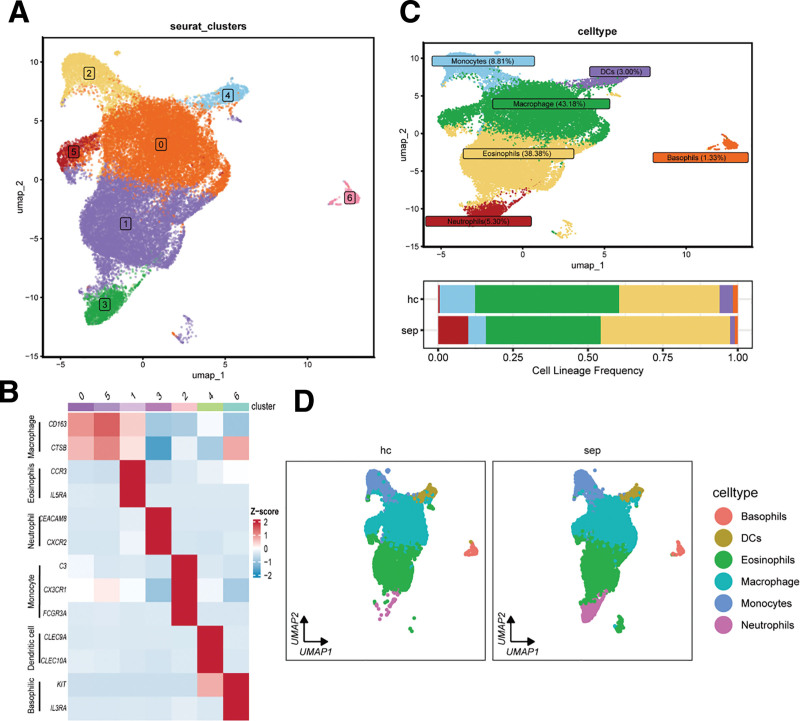
Differential analysis of myeloid cells in peripheral blood between healthy individuals and sepsis patients. (A) UMAP plot showing the distribution of myeloid cell subsets in peripheral blood from healthy individuals and sepsis patients. (B) Heatmap of marker gene expression used for annotating different myeloid cell subsets. (C) UMAP visualization of annotated myeloid cell subsets and bar plots showing the proportion distribution of each myeloid cell subset in healthy individuals and sepsis patients. (D) UMAP plot showing differences in the distribution of different myeloid cell types in peripheral blood between sepsis patients and healthy individuals.

Myeloid cells were subclustered into 7 subsets (Fig. 2A). These subsets were defined by canonical markers: Macrophages (CD163, CTSB), Eosinophils (CCR3, IL5RA), Neutrophils (CEACAM8, CXCR2), Monocytes (C3, CX3CR1, FCGR3A), Dendritic cells (CLEC9A, CLEC10A), and Basophils (KIT, IL3RA) (Fig. 2B). Annotated proportions of these subsets were macrophages (43.18%), eosinophils (38.38%), neutrophils (5.30%), monocytes (8.81%), dendritic cells (3.00%), and basophils (1.33%) (Fig. 2C). Sepsis patients had significantly higher neutrophil proportions relative to healthy controls (Fig. 2C and D), indicating a pivotal role for neutrophils in sepsis pathogenesis.

### 3.3. Identification and functional analysis of neutrophil-associated DEGs

**Figure 3.**
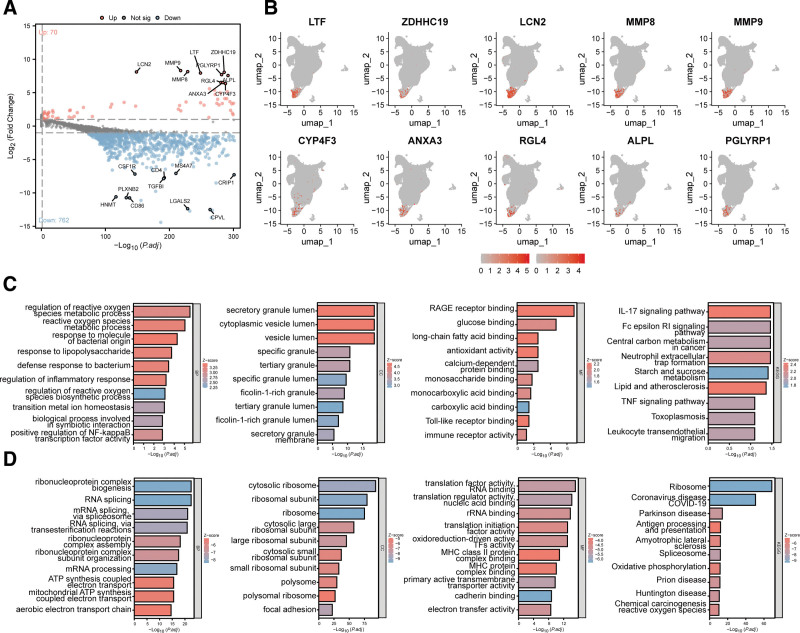
Identification of DEGs in peripheral blood neutrophils from healthy individuals and sepsis patients. (A) Volcano plot showing DEGs in neutrophils between healthy individuals and sepsis patients. (B) UMAP visualization showing expression of marker genes in neutrophil subclusters. (C) Results of functional enrichment analysis for upregulated neutrophil DEGs. (D) Results of functional enrichment analysis for downregulated neutrophil DEGs. DEGs = differentially expressed genes.

To investigate the roles of neutrophils in sepsis, we analyzed DEGs between neutrophils and other cell types. Using screening criteria (|logFC|>1 and *P* < .05), we identified 70 upregulated and 762 downregulated neutrophil-associated DEGs (Fig. 3A). UMAP visualization depicts the expression distributions of the top 10 highly expressed genes (Fig. 3B). To further explore the potential biological functions of these DEGs, we performed enrichment analyses on upregulated and downregulated DEGs, respectively. Enrichment analyses revealed that upregulated DEGs primarily participate in the reactive oxygen species metabolic process, response to molecules of bacterial origin, and defense response to bacterium (biological process, BP); are localized to the secretory granule lumen, cytoplasmic vesicle lumen, and specific granule (cellular component, CC); and exhibit RAGE receptor binding, glucose binding, long-chain fatty acid binding, and antioxidant activity (molecular function, MF). KEGG analysis showed significant enrichment in the IL-17 signaling pathway, Fc epsilon RI signaling pathway, NETosis and Leukocyte transendothelial migration (all *P* < .05; Fig. 3C). Downregulated DEGs were enriched in the ribonucleoprotein complex biogenesis, RNA splicing, and mRNA splicing via spliceosome (BP); cytosolic ribosome, ribosomal subunit, and cytosolic large ribosomal subunit (CC); and translation factor activity, RNA binding, translation regulator activity, nucleic acid binding, and MHC class II protein complex binding (MF). KEGG analysis indicated significant enrichment in the Ribosome, Antigen processing and presentation, and Spliceosome pathways (all *P* < .05; Fig. 3D). These findings demonstrate that neutrophil-upregulated DEGs participate in activating inflammatory responses, thereby promoting sepsis pathogenesis.

### 3.4. Screening of hub neutrophil-associated genes in sepsis

**Figure 4.**
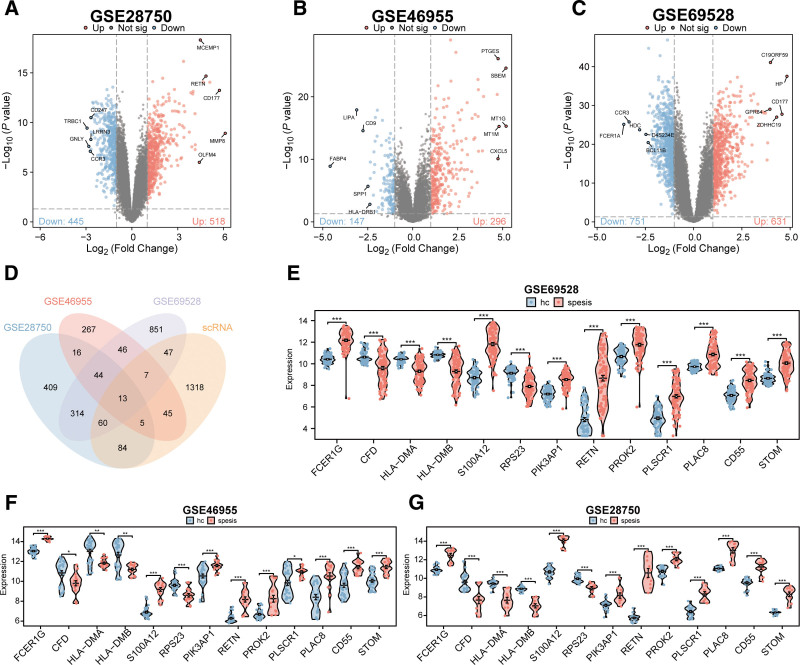
Screening of hub neutrophil-associated genes in sepsis. (A–C) Volcano plots showing expression profiles of DEGs between sepsis patients and healthy controls in 3 GEO datasets, respectively. (D) Venn diagram illustrating the intersection of overlapping genes between neutrophil DEGs and DEGs from the 3 datasets. (E–G) Violin plots showing expression level differences of the intersecting genes between sepsis patients and healthy controls in the 3 datasets. DEGs = differentially expressed genes, GEO = Gene Expression Omnibus.

To further explore which DEGs are key drivers of sepsis, we screened DEGs in blood samples between sepsis patients and healthy controls across 3 cohorts (GSE28750, GSE46955, and GSE69528) using the criteria |logFC| > 1 and *P* < .05. These analyses identified 445 downregulated and 518 upregulated genes in GSE28750 (Fig. 4A), 147 downregulated and 296 upregulated genes in GSE46955 (Fig. 4B), and 751 downregulated and 631 upregulated genes in GSE69528 (Fig. 4C). Further intersection of DEGs from the different cohorts with neutrophil-associated DEGs revealed that RETN, S100A12, STOM, PLAC8, CFD, PLSCR1, HLA-DMB, HLA-DMA, CD55, FCER1G, PROK2, PIK3AP1, and RPS23 are key genes (Fig. 4D). Among these, FCER1G, S100A12, PIK3AP1, RETN, PROK2, PLSCR1, PLAC8, CD55, and STOM were significantly upregulated in sepsis patients relative to healthy controls, while CFD, HLA-DMB, HLA-DMA, and RPS23 were significantly downregulated (all *P* < .05; Fig. 4E–G).

### 3.5. Construction of diagnostic models via 101 ML algorithms

**Figure 5.**
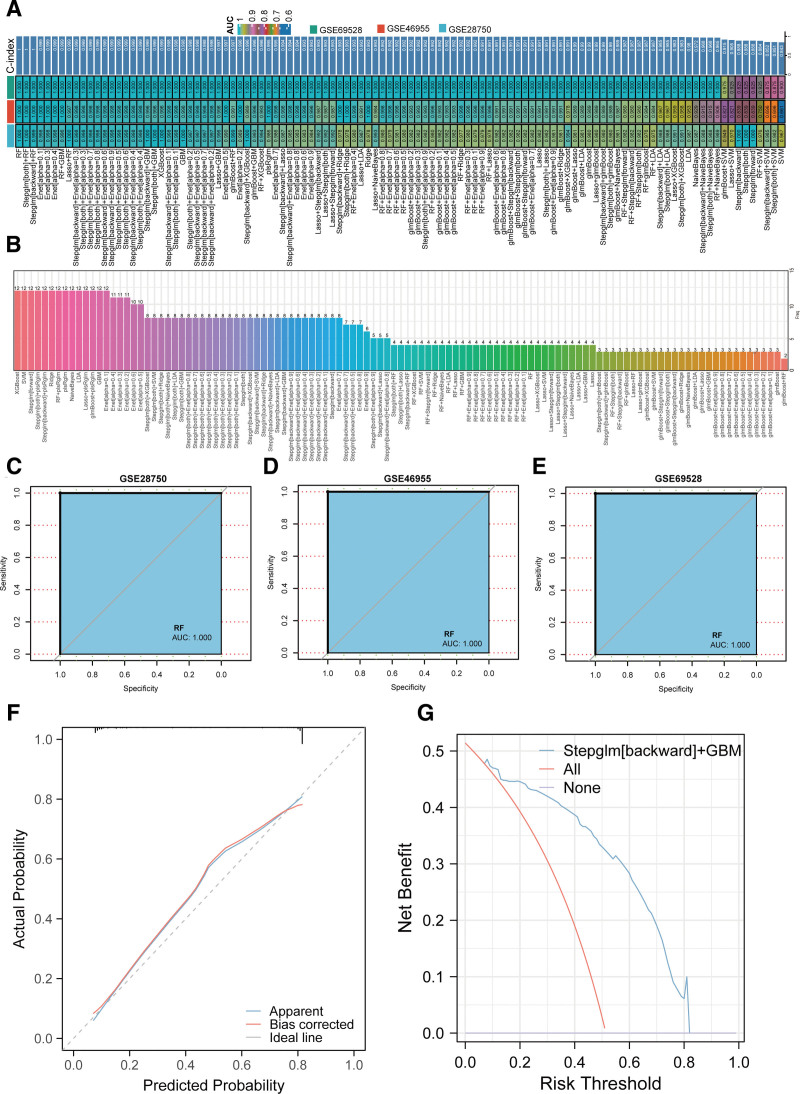
Construction and validation of sepsis diagnostic models via 113 machine learning algorithms. (A) Heatmap showing the mean C-index of diagnostic models constructed using 113 machine learning algorithms. (B) Bar plot showing the number of genes used in the 113 algorithms-based models. (C), (D), (E) ROC curves for the Random Forest (RF) model in the 3 cohorts. (F) Calibration curve for the RF model. (G) DCA of the RF model. DCA = decision curve analysis, RF = Random Forest, ROC = receiver operator characteristic.

ML facilitates the construction of precise and effective models for predicting sepsis. Therefore, with the GSE69528 cohort as the training set and the GSE46955 and GSE28750 cohorts as validation sets, we incorporated the 13 intersecting genes to build diagnostic models via 113 ML algorithms. Results indicated that the RF model, built using S100A12, PIK3AP1, HLA__DMB, and RETN, was identified as the optimal model owing to its highest mean C-index and fewer genes (Fig. 5A and B). Receiver operator characteristic curve analysis yielded AUC values of 1 for the RF model across all 3 cohorts (Fig. 5C–E). Calibration curve results showed that the RF model exhibited good accuracy (Fig. 5F). Finally, DCA results demonstrated that the RF model provided favorable clinical benefit (Fig. 5G).

### 3.6. S100A12 is a key variable in sepsis diagnosis

**Figure 6.**
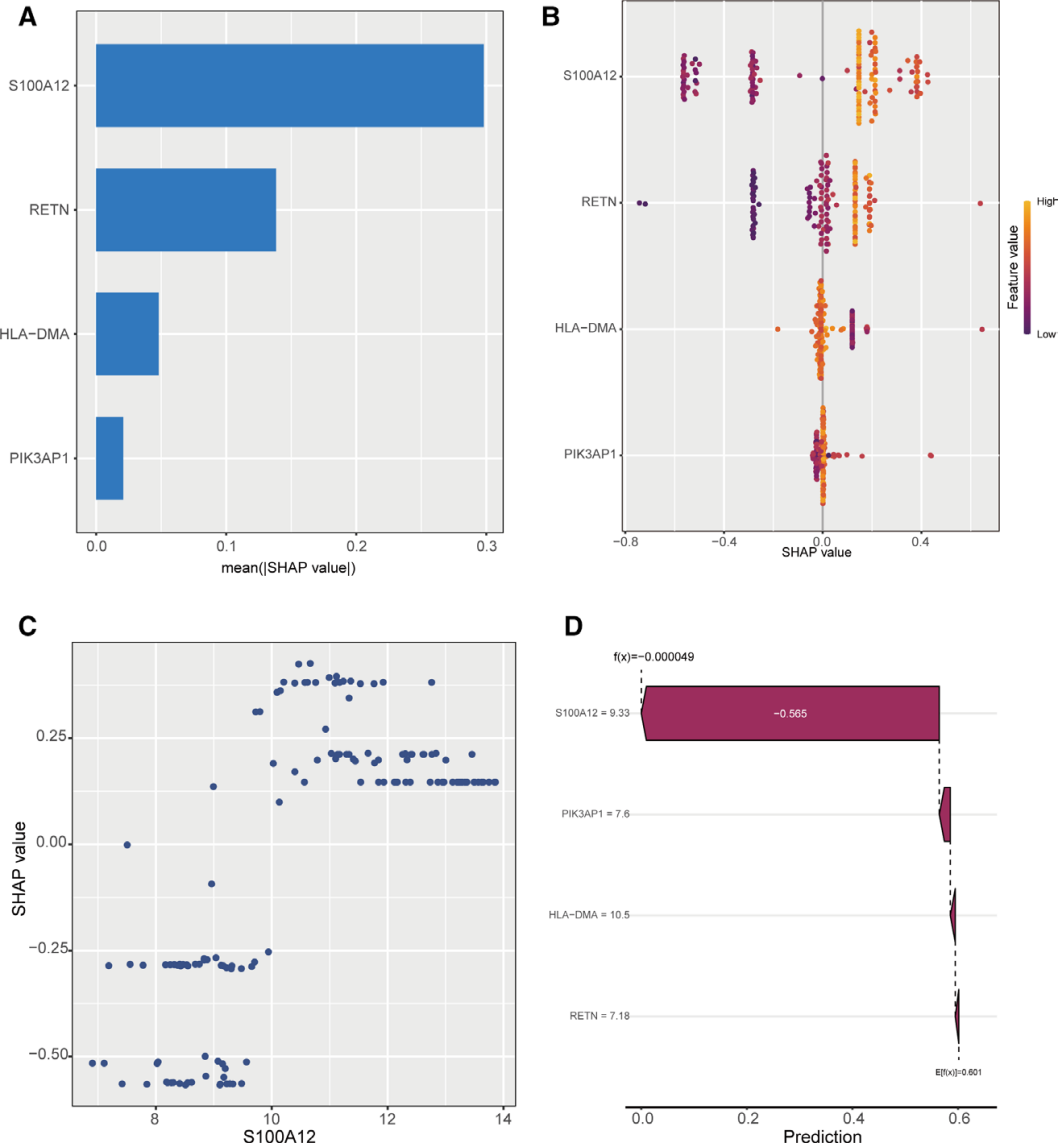
SHAP analysis of the sepsis diagnostic model. (A) SHAP bar plot of variable importance. (B) SHAP beeswarm plot. (C) SHAP dependence plot. (D) SHAP waterfall plot for patient GSM1702889. SHAP = Shapley additive explanations.

To further explore the influence of different variables within the RF model on sepsis prediction, we interpreted the RF model using SHAP analysis. The summary plot indicated that the S100A12 feature exhibited the largest mean absolute SHAP value, signifying its most substantial overall impact on the model’s predictions, while the PIK3AP1 feature showed the smallest mean absolute SHAP value, indicating its relatively weaker overall contribution. The mean absolute SHAP values for the RETN and HLA-DMA features were intermediate (Fig. 6A). The dependency plot revealed that as the feature value of S100A12 increased, the SHAP value increased accordingly, meaning that higher S100A12 expression exerted a greater positive influence on the prediction results (Fig. 6B). Furthermore, the SHAP values for S100A12 displayed substantial fluctuation across samples and were predominantly positive, further suggesting that this feature generally elevates the model’s predicted value in most cases (Fig. 6C). Finally, when analyzing data from a representative patient, Figure 6D shows the relationship between the model’s predicted value and feature SHAP values, demonstrating that S100A12 exerted the greatest contribution within the model. Therefore, S100A12 may serves as a linchpin in the accurate identification of sepsis.

### 3.7. Exploration of potential drugs targeting S100A12

**Figure 7.**
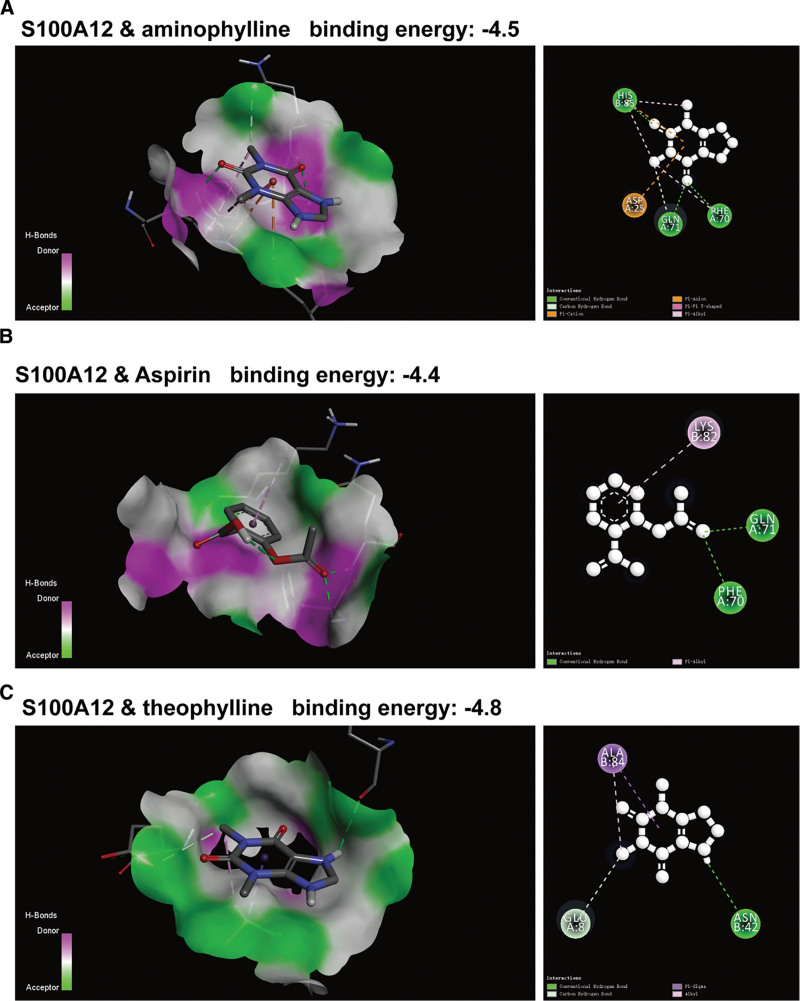
Screening of potential targeted drugs for S100A12. (A) Schematic diagrams of 3D (left) and 2D (right) binding between S100A12 and aminophylline. (B) Schematic diagrams of 3D (left) and 2D (right) binding between S100A12 and Aspirin. (C) Schematic diagrams of 3D (left) and 2D (right) binding between S100A12 and theophylline.

Through the analysis of exploring potential drugs targeting S100A12, the results show that 3 compounds, namely aminophylline, Aspirin and theophylline, have relatively high binding capacity with S100A12 protein, with their binding energies being −4.5, −4.4 and −4.8 respectively (Fig. 7A–C). This indicates that these 3 compounds may be potential drugs for the treatment of sepsis.

## 4. Discussion

Sepsis diagnosis and treatment currently grapple with the formidable challenges of a substantial complication burden and high lethality, with the dearth of robust biomarkers standing as a central impediment to precision medicine.^[24]^ In this investigation, peripheral blood single-cell profiling of 4 sepsis patients and 5 healthy controls uncovered a marked elevation in the neutrophil proportion within the myeloid cell compartment of sepsis patients. Subsequent integration of single-cell and bulk-RNA sequencing data, coupled with the implementation of a ML framework, led to the development of a RF model constructed with 4 genes that exhibited favorable diagnostic performance for sepsis. Finally, SHAP analysis highlighted S100A12 as a pivotal biomarker for sepsis.

This study identified 13 DEGs, all of which are potential key drivers of sepsis pathogenesis. On one hand, the IL-17 signaling pathway, FcεRI signaling pathway, neutrophil extracellular trap (NET) formation, and leukocyte transendothelial migration pathway are integral to core processes of the immune response: the IL-17 pathway mediates pro-inflammatory signal amplification^[25]^; the FcεRI pathway is linked to allergy-like inflammatory responses^[26]^; NETosis facilitates pathogen clearance and inflammation amplification^[27]^; and leukocyte transendothelial migration represents a critical step in immune cell recruitment.^[28,29]^ The activation status of these pathways, along with their potential association with sepsis diagnostic efficacy and prognostic assessment, is emerging as a focus of current research. On the other hand, biological processes involving these key genes – including ribosome-mediated protein synthesis, regulation of immune recognition via antigen processing and presentation, and fine-tuned regulation of gene expression by the spliceosome – all influence the onset and progression of sepsis by maintaining homeostasis of the systemic inflammatory response and immune function.^[30]^ Dysregulation of these pathways and processes may disrupt the balance of the immune regulatory network, thereby inducing and propelling the pathological progression of sepsis, offering novel candidate targets for deciphering the molecular mechanisms of sepsis.

Previous investigations have sought to identify sepsis-associated regulatory genes using ML techniques: Gong et al preliminarily identified SCAMP5 as a diagnostic biomarker implicated in sepsis progression via the regulation of endoplasmic reticulum stress, achieved by integrating ML algorithms with bioinformatics analysis^[31]^; Diao et al employed ML to validate the clinical utility of a platelet-related gene-based model for guiding early treatment decisions in septic patients.^[32]^ In this study, through systematic screening within a ML framework, we identified a RF diagnostic model constructed with S100A12, PIK3AP1, HLA-DMB, and RETN that exhibited exceptional predictive performance, achieving AUC values of 1.0 in both training and validation cohorts. Calibration curve and DCA further corroborated the model’s accuracy and clinical applicability, suggesting that this model represents a robust tool for sepsis diagnosis.

Furthermore, SHAP analysis of the ML model revealed that the S100A12 feature exhibited the largest mean absolute SHAP value, indicating its most substantial contribution to the model’s predictive performance and suggesting its superior clinical application potential among the candidate genes. S100A12, a member of the S100 family, functions as a key Ca²^+^-binding pro-inflammatory protein, whose regulatory roles in various pathological processes have attracted substantial attention.^[33–35]^ This molecule contributes to amplifying the inflammatory cascade through crosstalk and interactions with multiple host immune signaling pathways: on one hand, it may trigger cytokine storms by activating downstream pathways for pro-inflammatory cytokine release^[36]^; on the other hand, its mediation of immune dysregulation may disrupt pro-inflammatory and anti-inflammatory homeostasis, thereby inducing systemic inflammatory response syndrome and ultimately promoting the development of multi-organ failure.^[37]^ Clinical studies have demonstrated that aberrant S100A12 expression is widely implicated in diverse inflammatory diseases: in patients with rheumatoid arthritis, its high expression in synovial tissue is closely correlated with the severity of synovial inflammation and bone destruction; in patients with inflammatory bowel disease, S100A12 levels are significantly elevated in intestinal mucosa and serum, and positively correlated with intestinal inflammatory activity^[38]^; in cardiovascular diseases, S100A12 contributes to the formation and destabilization of atherosclerotic plaques by activating a pro-inflammatory phenotype in vascular endothelial cells.^[39]^ These studies collectively highlight S100A12’s potential clinical value as a sensitive biomarker for systemic inflammatory responses across multiple immune-related diseases. Although molecules such as MMP9 and PRTN3 have also been linked to sepsis prognosis, current investigations into these molecules in sepsis remain limited. In contrast, the mechanistic role of S100A12 in sepsis immune regulation is more clearly defined, meriting further in-depth investigation of its clinical translational potential as a diagnostic biomarker and potential therapeutic target.

Previous studies have shown that in the treatment of sepsis, albumin not only effectively expands intravascular volume compared with traditional crystalloids, but also modulates the inflammatory network by downregulating inflammatory mediator levels, regulating vascular permeability, and alleviating oxidative stress. However, clinical evidence has not reached a consensus on whether albumin administration can improve overall mortality in septic patients.^[40–43]^ Given the critical role of S100A12 in immune responses and the potential anti-inflammatory mechanisms of albumin, it is hypothesized that albumin may exert anti-inflammatory effects by downregulating S100A12 levels in septic patients. Nevertheless, the specific association between albumin and S100A12 regulation requires further validation through clinical sample analysis and mechanistic experiments.

This study has certain limitations. First, the biological mechanisms of S100A12 in sepsis have not been validated via in vitro or in vivo experiments; its immunoregulatory roles warrant further validation using animal models or serum samples, a priority for our subsequent research phase. We will prospectively collect biological samples from sepsis patients at our institution and integrate them with real-time dynamic clinical data to validate the accuracy of the RF model in subsequent studies. Nevertheless, through bioinformatics analysis, this study has identified key genes and pathways in sepsis and established S100A12 as an independent risk factor for prognosis in sepsis, highlighting its critical role in sepsis progression and providing new insights into targeted interventions.

## Author contributions

**Conceptualization:** Xiaoping Huang, Zibo Yu.

**Data curation:** Xiaoping Huang.

**Formal analysis:** Xiaoping Huang, Zhifang Zhuo.

**Investigation:** Xiaoping Huang, Zibo Yu.

**Resources:** Xiaoping Huang, Zibo Yu, Zhifang Zhuo.

**Software:** Zhifang Zhuo.

**Supervision:** Zhifang Zhuo.

**Validation:** Zibo Yu, Zhifang Zhuo.

**Writing – original draft:** Xiaoping Huang, Zibo Yu.

**Writing – review & editing:** Zhifang Zhuo.

## Supplementary Material


