# Supplementary material for: S100A12 as a key biomarker in a neutrophil-associated gene prediction model for sepsis diagnosis
Source: Medicine (Baltimore). 2025 Nov 21;104(47):e46140. doi: 10.1097/MD.0000000000046140 (PMC12643676; doi:10.1097/MD.0000000000046140)
Supplement: Supplementary file 1 [file medi-104-e46140-s001.docx]

Supplementary table 1. The clinical characteristic data of the GEO cohorts in this study.

| ID | cohort | group |
| --- | --- | --- |
| GSM1702888 | GSE69528 | healthy |
| GSM1702889 | GSE69528 | healthy |
| GSM1702890 | GSE69528 | healthy |
| GSM1702891 | GSE69528 | healthy |
| GSM1702892 | GSE69528 | healthy |
| GSM1702893 | GSE69528 | healthy |
| GSM1702894 | GSE69528 | healthy |
| GSM1702895 | GSE69528 | healthy |
| GSM1702896 | GSE69528 | healthy |
| GSM1702897 | GSE69528 | healthy |
| GSM1702898 | GSE69528 | healthy |
| GSM1702899 | GSE69528 | healthy |
| GSM1702900 | GSE69528 | healthy |
| GSM1702901 | GSE69528 | healthy |
| GSM1702902 | GSE69528 | healthy |
| GSM1702903 | GSE69528 | healthy |
| GSM1702904 | GSE69528 | healthy |
| GSM1702905 | GSE69528 | healthy |
| GSM1702906 | GSE69528 | healthy |
| GSM1702907 | GSE69528 | healthy |
| GSM1702908 | GSE69528 | healthy |
| GSM1702909 | GSE69528 | healthy |
| GSM1702910 | GSE69528 | healthy |
| GSM1702911 | GSE69528 | healthy |
| GSM1702912 | GSE69528 | healthy |
| GSM1702913 | GSE69528 | healthy |
| GSM1702914 | GSE69528 | healthy |
| GSM1702915 | GSE69528 | healthy |
| GSM1702916 | GSE69528 | healthy |
| GSM1702917 | GSE69528 | healthy |
| GSM1702918 | GSE69528 | healthy |
| GSM1702919 | GSE69528 | healthy |
| GSM1702920 | GSE69528 | healthy |
| GSM1702921 | GSE69528 | healthy |
| GSM1702922 | GSE69528 | healthy |
| GSM1702923 | GSE69528 | healthy |
| GSM1702924 | GSE69528 | healthy |
| GSM1702925 | GSE69528 | healthy |
| GSM1702926 | GSE69528 | healthy |
| GSM1702927 | GSE69528 | healthy |
| GSM1702928 | GSE69528 | healthy |
| GSM1702929 | GSE69528 | healthy |
| GSM1702930 | GSE69528 | healthy |
| GSM1702931 | GSE69528 | healthy |
| GSM1702932 | GSE69528 | healthy |
| GSM1702933 | GSE69528 | healthy |
| GSM1702934 | GSE69528 | healthy |
| GSM1702935 | GSE69528 | healthy |
| GSM1702936 | GSE69528 | healthy |
| GSM1702937 | GSE69528 | healthy |
| GSM1702938 | GSE69528 | healthy |
| GSM1702939 | GSE69528 | healthy |
| GSM1702940 | GSE69528 | healthy |
| GSM1702941 | GSE69528 | healthy |
| GSM1702942 | GSE69528 | healthy |
| GSM1702943 | GSE69528 | sepsis |
| GSM1702944 | GSE69528 | sepsis |
| GSM1702945 | GSE69528 | sepsis |
| GSM1702946 | GSE69528 | sepsis |
| GSM1702947 | GSE69528 | sepsis |
| GSM1702948 | GSE69528 | sepsis |
| GSM1702949 | GSE69528 | sepsis |
| GSM1702950 | GSE69528 | sepsis |
| GSM1702951 | GSE69528 | sepsis |
| GSM1702952 | GSE69528 | sepsis |
| GSM1702953 | GSE69528 | sepsis |
| GSM1702954 | GSE69528 | sepsis |
| GSM1702955 | GSE69528 | sepsis |
| GSM1702956 | GSE69528 | sepsis |
| GSM1702957 | GSE69528 | sepsis |
| GSM1702958 | GSE69528 | sepsis |
| GSM1702959 | GSE69528 | sepsis |
| GSM1702960 | GSE69528 | sepsis |
| GSM1702961 | GSE69528 | sepsis |
| GSM1702962 | GSE69528 | sepsis |
| GSM1702963 | GSE69528 | sepsis |
| GSM1702964 | GSE69528 | sepsis |
| GSM1702965 | GSE69528 | sepsis |
| GSM1702966 | GSE69528 | sepsis |
| GSM1702967 | GSE69528 | sepsis |
| GSM1702968 | GSE69528 | sepsis |
| GSM1702969 | GSE69528 | sepsis |
| GSM1702970 | GSE69528 | sepsis |
| GSM1702971 | GSE69528 | sepsis |
| GSM1702972 | GSE69528 | sepsis |
| GSM1702973 | GSE69528 | sepsis |
| GSM1702974 | GSE69528 | sepsis |
| GSM1702975 | GSE69528 | sepsis |
| GSM1702976 | GSE69528 | sepsis |
| GSM1702977 | GSE69528 | sepsis |
| GSM1702978 | GSE69528 | sepsis |
| GSM1702979 | GSE69528 | sepsis |
| GSM1702980 | GSE69528 | sepsis |
| GSM1702981 | GSE69528 | sepsis |
| GSM1702982 | GSE69528 | sepsis |
| GSM1702983 | GSE69528 | sepsis |
| GSM1702984 | GSE69528 | sepsis |
| GSM1702985 | GSE69528 | sepsis |
| GSM1702986 | GSE69528 | sepsis |
| GSM1702987 | GSE69528 | sepsis |
| GSM1702988 | GSE69528 | sepsis |
| GSM1702989 | GSE69528 | sepsis |
| GSM1702990 | GSE69528 | sepsis |
| GSM1702991 | GSE69528 | sepsis |
| GSM1702992 | GSE69528 | sepsis |
| GSM1702993 | GSE69528 | sepsis |
| GSM1702994 | GSE69528 | sepsis |
| GSM1702995 | GSE69528 | sepsis |
| GSM1702996 | GSE69528 | sepsis |
| GSM1702997 | GSE69528 | sepsis |
| GSM1702998 | GSE69528 | sepsis |
| GSM1702999 | GSE69528 | sepsis |
| GSM1703000 | GSE69528 | sepsis |
| GSM1703001 | GSE69528 | sepsis |
| GSM1703002 | GSE69528 | sepsis |
| GSM1703003 | GSE69528 | sepsis |
| GSM1703004 | GSE69528 | sepsis |
| GSM1703005 | GSE69528 | sepsis |
| GSM1703006 | GSE69528 | sepsis |
| GSM1703007 | GSE69528 | sepsis |
| GSM1703008 | GSE69528 | sepsis |
| GSM1703009 | GSE69528 | sepsis |
| GSM1703010 | GSE69528 | sepsis |
| GSM1703011 | GSE69528 | sepsis |
| GSM1703012 | GSE69528 | sepsis |
| GSM1703013 | GSE69528 | sepsis |
| GSM1703014 | GSE69528 | sepsis |
| GSM1703015 | GSE69528 | sepsis |
| GSM1703016 | GSE69528 | sepsis |
| GSM1703017 | GSE69528 | sepsis |
| GSM1703018 | GSE69528 | sepsis |
| GSM1703019 | GSE69528 | sepsis |
| GSM1703020 | GSE69528 | sepsis |
| GSM1703021 | GSE69528 | sepsis |
| GSM1703022 | GSE69528 | sepsis |
| GSM1703023 | GSE69528 | sepsis |
| GSM1703024 | GSE69528 | sepsis |
| GSM1703025 | GSE69528 | sepsis |
| GSM1141941 | GSE46955 | healthy |
| GSM1141942 | GSE46955 | healthy |
| GSM1141943 | GSE46955 | healthy |
| GSM1141944 | GSE46955 | healthy |
| GSM1141945 | GSE46955 | sepsis |
| GSM1141946 | GSE46955 | sepsis |
| GSM1141947 | GSE46955 | healthy |
| GSM1141948 | GSE46955 | healthy |
| GSM1141949 | GSE46955 | healthy |
| GSM1141950 | GSE46955 | healthy |
| GSM1141951 | GSE46955 | sepsis |
| GSM1141952 | GSE46955 | sepsis |
| GSM1141953 | GSE46955 | healthy |
| GSM1141954 | GSE46955 | healthy |
| GSM1141955 | GSE46955 | healthy |
| GSM1141956 | GSE46955 | healthy |
| GSM1141957 | GSE46955 | sepsis |
| GSM1141958 | GSE46955 | sepsis |
| GSM1141959 | GSE46955 | healthy |
| GSM1141960 | GSE46955 | healthy |
| GSM1141961 | GSE46955 | healthy |
| GSM1141962 | GSE46955 | healthy |
| GSM1141963 | GSE46955 | healthy |
| GSM1141964 | GSE46955 | healthy |
| GSM1141965 | GSE46955 | healthy |
| GSM1141966 | GSE46955 | healthy |
| GSM1141967 | GSE46955 | sepsis |
| GSM1141968 | GSE46955 | sepsis |
| GSM1141969 | GSE46955 | healthy |
| GSM1141970 | GSE46955 | healthy |
| GSM1141971 | GSE46955 | sepsis |
| GSM1141972 | GSE46955 | sepsis |
| GSM1141973 | GSE46955 | healthy |
| GSM1141974 | GSE46955 | healthy |
| GSM1141975 | GSE46955 | sepsis |
| GSM1141976 | GSE46955 | sepsis |
| GSM1141977 | GSE46955 | healthy |
| GSM1141978 | GSE46955 | healthy |
| GSM1141979 | GSE46955 | sepsis |
| GSM1141980 | GSE46955 | sepsis |
| GSM1141981 | GSE46955 | healthy |
| GSM1141982 | GSE46955 | healthy |
| GSM1141983 | GSE46955 | sepsis |
| GSM1141984 | GSE46955 | sepsis |
| GSM712478 | GSE28750 | sepsis |
| GSM712479 | GSE28750 | sepsis |
| GSM712480 | GSE28750 | sepsis |
| GSM712481 | GSE28750 | sepsis |
| GSM712482 | GSE28750 | sepsis |
| GSM712483 | GSE28750 | sepsis |
| GSM712484 | GSE28750 | sepsis |
| GSM712485 | GSE28750 | sepsis |
| GSM712486 | GSE28750 | sepsis |
| GSM712487 | GSE28750 | sepsis |
| GSM712488 | GSE28750 | healthy |
| GSM712489 | GSE28750 | healthy |
| GSM712490 | GSE28750 | healthy |
| GSM712491 | GSE28750 | healthy |
| GSM712492 | GSE28750 | healthy |
| GSM712493 | GSE28750 | healthy |
| GSM712494 | GSE28750 | healthy |
| GSM712495 | GSE28750 | healthy |
| GSM712496 | GSE28750 | healthy |
| GSM712497 | GSE28750 | healthy |
| GSM712498 | GSE28750 | healthy |
| GSM712499 | GSE28750 | healthy |
| GSM712500 | GSE28750 | healthy |
| GSM712501 | GSE28750 | healthy |
| GSM712502 | GSE28750 | healthy |
| GSM712503 | GSE28750 | healthy |
| GSM712504 | GSE28750 | healthy |
| GSM712505 | GSE28750 | healthy |
| GSM712506 | GSE28750 | healthy |
| GSM712507 | GSE28750 | healthy |
